# The Cognitive Drivers of Compulsive Eating Behavior

**DOI:** 10.3389/fnbeh.2018.00338

**Published:** 2019-01-17

**Authors:** Naomi Kakoschke, Esther Aarts, Antonio Verdejo-García

**Affiliations:** ^1^Monash Institute for Cognitive and Clinical Neurosciences (MICCN), School of Psychological Sciences, Monash University, Melbourne, VIC, Australia; ^2^Donders Centre for Cognitive Neuroimaging, Donders Institute for Brain, Cognition and Behaviour, Radboud University, Nijmegen, Netherlands

**Keywords:** compulsivity, cognitive functioning, eating behavior, obesity, bulimia nervosa, binge eating, food addiction

## Abstract

Compulsivity is a central feature of obsessive-compulsive and addictive disorders, which share considerable overlap with excessive eating in terms of repetitive behavior despite negative consequences. Excessive eating behavior is characteristic of several eating-related conditions, including eating disorders [bulimia nervosa (BN), binge eating disorder (BED)], obesity, and food addiction (FA). Compulsivity is proposed to be driven by four distinct cognitive components, namely, contingency-related cognitive flexibility, task/attentional set-shifting, attentional bias/disengagement and habit learning. However, it is unclear whether repetitive behavior in eating-related conditions is underpinned by deficits in these cognitive components. The current mini-review synthesizes the available evidence for performance on compulsivity-related cognitive tasks for each cognitive domain among populations with excessive eating behavior. In three of the four cognitive domains, i.e., set-shifting, attentional bias and habit learning, findings were mixed. Evidence more strongly pointed towards impaired contingency-related cognitive flexibility only in obesity and attentional bias/disengagement deficits only in obesity and BED. Overall, the findings of the reviewed studies support the idea that compulsivity-related cognitive deficits are common across a spectrum of eating-related conditions, although evidence was inconsistent or lacking for some disorders. We discuss the theoretical and practical importance of these results, and their implications for our understanding of compulsivity in eating-related conditions.

## Introduction

Compulsivity is defined as “the performance of repetitive, unwanted and functionally impairing overt or covert behaviors without adaptive function, performed in a habitual or stereotyped fashion, either according to rigid rules or as a means of avoiding perceived negative consequences” (Fineberg et al., 2014, p. 70). Behavioral patterns of compulsive eating, defined as repetitive bouts, without homeostatic function, with adverse consequences, and as ways to relieve stress, are common across several eating-related conditions (Moore et al., 2017). These include: (1) eating disorders such as bulimia nervosa (BN) and binge eating disorder (BED); (2) obesity; and (3) food addiction (FA), which have very different diagnostic considerations (Table 1). However, is it important to acknowledge that the validity of FA is a highly debated and controversial concept within the scientific community (Ziauddeen and Fletcher, 2013; Hebebrand et al., 2014; Cullen et al., 2017). In this review article, we examine the cognitive underpinnings of this transdiagnostic compulsive eating phenotype. To do so, we adopt the four cognitive components of compulsivity proposed in the framework by Fineberg et al. (2014; i.e., cognitive flexibility, set-shifting, attentional bias/disengagement, and habit learning), and review studies that measured at least one component in adults with BN, BED, obesity or FA. To ensure timeliness, we only reviewed research published in the last 5 years (for reviews of earlier work in discrete domains see: Wu et al., 2014; Stojek et al., 2018).

**Table 1:** Clinical characteristics of bulimia nervosa (BN), binge eating disorder (BED), obesity, and food addiction (FA).

Bulimia nervosa (BN)	Binge eating disorder (BED)	Obesity	Food addiction (FA)
**Recurrent episodes of binge eating (BE)** characterized by: (a) eating within a 2 h period of time an amount of food larger than what most people would eat in a similar period of time under similar circumstances; and (b) a sense of lack of control overeating during the episode **Recurrent inappropriate compensatory behavior** in order to prevent weight gain, such as self-induced vomiting, misuse of laxatives, diuretics, or other medications, fasting, or excessive exercise. The binge eating and inappropriate compensatory behaviors both occur, on average, at least once a week for 3 months. Self-evaluation is unduly influenced by body shape and weight. The disturbance does not occur exclusively during episodes of Anorexia Nervosa.	**Recurrent episodes of BE** characterized by: (a) eating within a 2 h period of time an amount of food larger than what most people would eat in a similar period of time under similar circumstances; and (b) a sense of lack of control overeating during the episode BE episodes are associated with three (or more) of the following cognitive symptoms: Eating much more rapidly than normal **Eating until feeling uncomfortably full** **Eating large amounts of food when not feeling physically hungry** Eating alone because of feeling embarrassed Feeling disgusted with oneself, depressed, or very guilty afterward **Marked distress regarding BE** BE occurs, on average, at least once a week for 3 months BE is not associated with the recurrent use of inappropriate compensatory behaviors (e.g., purging) and does not occur exclusively during the course of Bulimia Nervosa or Anorexia Nervosa.	Body mass index [(BMI) = body weight (kg)/height (m^2^) ≥30 BMI 30–39 = obese BMI ≥40 = morbidly obese Chronic overeating, i.e., excessive calorie intake relative to energy expenditure	**Consumed more than planned** (larger amount and for a longer period) Unable to cut down or stop Great deal of time spent Important activities given up or reduced **Use despite knowledge of physical/emotional consequences** Tolerance (increase in amount, decrease in effect) Withdrawal (symptoms, substance taken to relieve withdrawal) Craving or strong desire Failure in role obligation Use despite interpersonal/social consequences Use in physically hazardous situations

*Note: BN and BED symptoms defined according to DSM 5 diagnostic criteria (American Psychiatric Association, 2013). BMI categories defined according to the World Health Organisation (2017). FA symptoms defined according to the proposal by Gearhardt et al. (2016). Bold font denotes characteristics that fit a compulsive eating phenotype (i.e., repeated bouts, without adaptive–homeostatic function and/or driven by stress relief)*.

## Review of Findings

**Figure 1:**
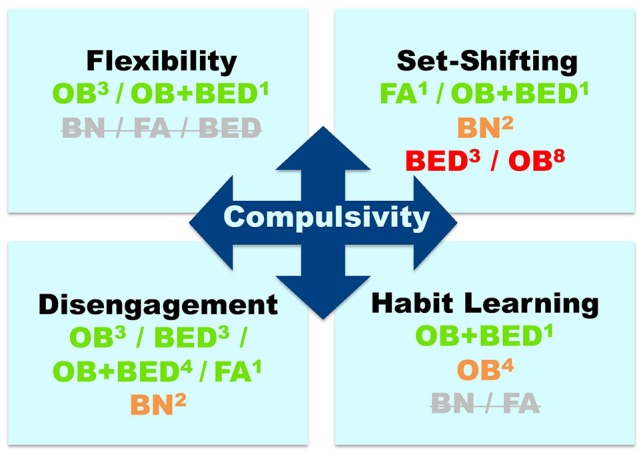
Evidence for compulsivity-related cognitive deficits across eating-related conditions: bulimia nervosa (BN), binge-eating disorder (BED), obesity (OB), and food addiction (FA). Colors indicate the direction of the evidence, namely, green: consistent evidence of deficits; orange: inconsistent evidence (approximately 50% of studies suggesting deficits/lack of deficits); red: negative evidence = no deficits (indicated by >60% of studies); Strikethrough gray: no available studies. Superscripts indicate the number of studies on each cognitive component and disorder.

In this section, we define each of the cognitive components of compulsivity and the tasks that measure them, and then review evidence of task performance in: (1) BN and BED; (2) obesity; (3) FA; and (4) overlapping conditions (e.g., obesity and BED; obesity and FA). Figure 1 displays a summary of the findings.

### Contingency-Related Cognitive Flexibility

This component refers to “impaired adaptation of behavior after negative feedback” (Fineberg et al., 2014). It has been posited that compulsivity arises from perseverating on a behavior that was once rewarded, but then becomes associated with negative consequences, indicating less cognitive flexibility. Contingency-related cognitive flexibility has frequently been measured using the probabilistic reversal learning task (PRLT; Cools et al., 2002; Clarke et al., 2005), which involves choosing between two stimuli and learning that one is usually rewarded (positive outcome), while the other is usually punished (negative outcome). The rule then changes and participants need to adapt their behavior in response to the outcome change.

Although no studies have examined this component in BN, BED alone or FA, cognitive flexibility deficits have been observed in obesity. Specifically, individuals with obesity showed more difficulty inhibiting a previously learnt behavioral rule indicated by increased perseverative errors on the Rule Shift Cards task (Spitoni et al., 2017). Women with obesity also showed reversal learning deficits specific to food, but not monetary cues (Zhang et al., 2014). Contradictory findings have also been reported, whereby participants with obesity showed impaired punishment, but not reward learning relative to healthy controls (Coppin et al., 2014; Banca et al., 2016), while obese participants with BED showed impaired reward, but not punishment learning relative to those without BED (Banca et al., 2016).

### Task/Attentional Set-Shifting

This component is defined as “impaired switching of attention between stimuli” (Fineberg et al., 2014). It involves frequent switching between sets of tasks or response types, which requires paying attention to multiple dimensions of stimuli. Of note, set-shifting is also contingency related, but it relies on stimulus-response sets rather than reward and punishment outcomes. The most common set-shifting measures were the Wisconsin Card Sorting Test (WCST) and the Trail Making Task Part-B (TMT-B), while the Intra-Dimensional/Extra-Dimensional set-shift task (Robbins et al., 1998) and the Task-Switching Paradigm (Steenbergen et al., 2015) were used less frequently. The WCST involves matching a card with specific features (e.g., color, shape) to one of four other cards using a “matching rule,” which changes over the course of the task. In the TMT-B, participants are asked to draw a line linking alternating numbers and letters (i.e., 1-A-2-B-3-C).

Most research on set-shifting has focused on eating disorders. Some studies found that set-shifting was not impaired in BN (Pignatti and Bernasconi, 2013), BED (Manasse et al., 2015), or sub-threshold BE symptoms (Kelly et al., 2013). However, Kelly et al. (2013) found that total number of binge episodes were positively correlated with perseverative errors on the WCST (i.e., poorer set-shifting). Furthermore, other studies found impaired set-shifting in patients diagnosed with BED or BN relative to healthy controls (Goddard et al., 2014; Aloi et al., 2015).

In obesity, studies examining set-shifting have produced inconsistent results. Specifically, some studies found no evidence of impaired performance (Chamberlain et al., 2015; Fagundo et al., 2016; Manasse et al., 2014; Schiff et al., 2016; Wu et al., 2016), while other studies found impaired set-shifting in participants with overweight or obesity relative to healthy controls (Gameiro et al., 2017; Steenbergen and Colzato, 2017) and eating disorder patients (Perpiñá et al., 2017). Studies have also shown impaired set-shifting in obese participants with BED, but not in those without (Banca et al., 2016), and obese participants with high, but not low FA symptoms (Rodrigue et al., 2018).

### Attentional Bias/Disengagement

This component entails “impaired shifting of mental sets away from stimuli” (Fineberg et al., 2014). Attentional bias involves the automatic orienting of attention towards certain stimuli; an aspect of selective attention (Cisler and Koster, 2010), while disengagement refers to an inability to direct/shift attention away from such stimuli, which may contribute to compulsive behavior *via* rigidity induced by disorder-relevant stimuli (Fineberg et al., 2014). Attentional bias is commonly measured with the Visual Probe Task (VPT), in which participants are instructed to respond to a dot that appears on the left or right side of a computer screen immediately following the presentation of a pair of stimuli, or the Emotional Stroop, in which participants are asked to name the ink color of a written word while ignoring its content.

Several studies have provided evidence of an attentional bias for unhealthy food cues in BN (Albery et al., 2016), BED (Sperling et al., 2017), or subthreshold BE symptoms (Popien et al., 2015), although one recent study found no evidence of attentional bias for unhealthy food in BED or BN relative to healthy weight controls (Lee et al., 2017). Some studies have also shown an attentional bias for unhealthy food in obese compared to healthy weight participants (Kemps et al., 2014; Bongers et al., 2015), while another study found no relationship between attentional bias toward food words and obesity-related indices (body mass index, BMI and abdominal fat; Janssen et al., 2017). Nevertheless, obese individuals with BED show a stronger attentional bias to unhealthy food cues than those without BED or normal-weight controls (Schag et al., 2013; Schmitz et al., 2014, 2015), and individuals with obesity and subthreshold BE symptoms showed more difficulty disengaging from such cues than those without BE (Deluchi et al., 2017). Participants with obesity and FA also had a larger attentional bias and more difficulty disengaging from unhealthy food cues relative to healthy weight controls without FA (Frayn et al., 2016).

### Habit Learning

This component involves “lack of sensitivity to goals or outcomes of actions” (Fineberg et al., 2014). Associative learning theories of instrumental behavior posit that actions are supported by two systems: a goal-directed and a habitual system (Balleine and Dickinson, 1998; de Wit and Dickinson, 2009). Compulsivity is hypothesized to arise from a shift away from goal-directed action toward habit due to an imbalance in these two underlying systems, i.e., an impaired goal-directed or overactive habit system. Evidence for an imbalance between these two systems can be tested with instrumental decision-making paradigms. In outcome devaluation tasks, participants have to refrain from responding to cues when the rewards associated with them have been devalued by selectively changing outcome contingencies as in the Slips-of-Action task (de Wit et al., 2012) or sensory-specific satiety (Balleine and Dickinson, 1998). The Two-Stage task uses a model-free/model-based reinforcement learning paradigm in which participants are instructed to make choices based on previously reinforced choices (model-free, “habit”-like) or future goal states (model-based, “goal-directed;” Daw et al., 2011).

Results from studies on habit learning in obesity are inconsistent. Specifically, two studies have shown that individuals with obesity were less sensitive to action outcomes, i.e., action control was shifted towards habitual control and away from goal-directed control, which suggests that these two systems are unbalanced (Horstmann et al., 2011; Janssen et al., 2017). In contrast, two other studies using the Slips-of-Action task found that participants with obesity did not make more slips-of-action than healthy weight participants (Dietrich et al., 2016; Watson et al., 2017). However, another study demonstrated that obese individuals with BED showed greater impairments in goal-directed (model-based) than habitual (model-free) responses than obese participants without BED or healthy-weight participants (Voon et al., 2015a).

## Discussion

Our review indicates some evidence of deficits across the four compulsivity-related cognitive processes among individuals with excessive eating-related problems. However, for most eating-related conditions (except for the overlapping condition, namely, obesity with BED) the data are inconclusive regarding impairments in the cognitive domains. These conflicting findings make it difficult to draw firm conclusions regarding the role of compulsivity-related cognitive deficits underlying problematic eating behavior across conditions. Nevertheless, the findings are first discussed for each compulsivity-related cognitive domain across the spectrum of eating-related problems. We then provide a conceptual discussion regarding the extent to which cognitive components related to compulsivity should be applied in the context of eating behavior, which is followed by an operational discussion of how we can move forward experimentally to advance our understanding of compulsivity-related cognitive functions.

The available research on contingency-related cognitive flexibility (i.e., reversal learning) shows a consistent pattern of results, namely, impaired reversal learning in obesity and BED. However, there were differences in terms of valence of impaired reversal learning (i.e., reward vs. punishment), which differed across conditions (i.e., obesity alone or obesity with BED). A potential explanation for the discrepant findings is that obese individuals with BED may be more likely to respond based on previously rewarded behaviors, while obese individuals without BED may be more likely to avoid responding based on previously punished behaviors (Banca et al., 2016). This idea is further supported by the finding of increased sensitivity to reward and enhanced risk taking in relation to reward expectation in obese individuals with BED, but not those without (Voon et al., 2015b). However, these findings do not align with the general view that BED is underpinned by negative reinforcement mechanisms (Vannucci et al., 2015). Nevertheless, it has been proposed that BED is characterized by generalized impairments in cognitive flexibility (Voon et al., 2015a). Thus, further studies are needed to unravel the role of reversal learning in obesity and BED. Finally, there was a lack of evidence for reversal learning in populations with BN or FA, and hence, the findings are limited to obese individuals with or without BED.

Within the domain of task/attentional set-shifting, studies also revealed mixed findings, which might be attributable to differences in sample composition (e.g., age and BMI) and methodology (i.e., self-reported vs. diagnosed BE; different cognitive tasks used to measure set-shifting ability). For example, the ID/ED task is proposed to measure multiple components of compulsivity, namely, reversal learning and set-shifting (Wildes et al., 2014), while the TMT-B measures only set-shifting ability. One possible explanation for the discrepant findings in the literature is that individuals with eating disorders or obesity might show deficits in some sub-components of set-shifting (e.g., engaging in vs. disengaging from a task-set), but not others (e.g., keeping the relevant task dimension online in working memory). Thus, the different facets involved in the various tasks used across studies may contribute to the contradictory results in this domain. In line with this idea, a recent meta-analysis demonstrated a small-to-medium effect size for inefficient set-shifting in BN, BED and obesity (Wu et al., 2014), which suggests that other factors may interact with set-shifting to predict compulsive eating behavior. Taken together, our review and the meta-analysis by Wu et al. (2014) suggest that set-shifting inefficiency is one compulsivity-related cognitive domain that may contribute to compulsive eating behavior.

The findings of this review also provide evidence for attentional bias/disengagement for disorder-specific cues, i.e., unhealthy food, in BED, obesity, and BED with obesity, although not all studies showed this effect, which is consistent with a recent review on attentional bias in BE-related disorders (Stojek et al., 2018). However, there was considerable variability in the tasks used to assess attentional bias, i.e., the Emotional Stroop or the VPT, the latter of which can distinguish between attentional bias and inability to disengage. Furthermore, the Stroop task requires executive functions other than attention, including inhibitory control (Balleine and Dickinson, 1998; de Wit and Dickinson, 2009), and thus, attentional bias may be linked to compulsive behavior more indirectly than the other cognitive components. Few studies assessed attentional bias/disengagement in BN or FA, which was also observed in the review by Stojek et al. (2018). Thus, future research should employ tasks that examine both attentional bias and disengagement from disorder specific stimuli across the spectrum of eating-related issues.

The tasks used to assess habit learning also demonstrated impairments in obesity and BED, although the studies in this domain were limited to these two eating-related populations. The finding that a propensity toward habit learning was shown with model-free vs. model-based and outcome devaluation tasks, but not the slips-of-action task indicates that these tasks may measure different aspects of habit learning. For example, behavior may be a consequence of an impaired goal-directed system or an overactive habit system, which can be distinguished using the Two-Stage task (Voon et al., 2015a). Moreover, the type of outcome devaluation in devaluation tasks matters. Due to possible obesity-related decreases in interoceptive sensitivity (Herbert and Pollatos, 2014), outcome devaluation *via* satiation (Horstmann et al., 2011; Janssen et al., 2017) might be less effective than outcome devaluation *via* instruction for overweight/obese individuals (Dietrich et al., 2016; Watson et al., 2017). While evidence for a propensity toward habit learning was more consistent in BED than obesity, more studies are needed before conclusions are drawn.

### Limitations and Future Research Directions

Our review highlights the emerging body of work on cognitive underpinnings, but well-established aspects of the compulsive eating phenotype, that still need to be incorporated in a cognitive model of compulsivity. Specifically, it is not clear how negative reinforcement mechanisms (i.e., emotional eating) or dietary restraint and related anxiety/stress, which are key drivers of compulsive eating in BN, BED and obesity, might interrelate with the cognitive components proposed by Fineberg et al. (2014). Research on habitual learning suggests that the balance between habit and goal-directed action control systems might depend upon factors such as stress (Schwabe and Wolf, 2011), while set-shifting deficits are modulated by anxiety (Billingsley-Marshall et al., 2013), and attentional bias toward unhealthy food cues is moderated by emotional eating (Hepworth et al., 2010). Future studies should test whether emotional eating and stress/anxiety interact with compulsivity-related cognitive deficits to predict the emergence of pathological compulsive eating.

Theoretically, the findings of the current review also have implications for our current understanding of eating problems. Specifically, eating disorders, namely, BN and BED, are considered psychiatric disorders, whereas obesity is typically considered a physiological condition. Our finding that eating disorders and obesity share common cognitive alterations related to compulsivity is consistent with the idea that obesity can be better conceptualized as a biobehavioral disorder characterized by physiological as well as neural, cognitive and behavioral problems that are present across the spectrum of eating disorders (Volkow and Wise, 2005; Wilson, 2010). However, it should be noted that obesity is a highly heterogeneous disorder, and that the “compulsive eating” phenotype, characterized by repetitive bouts, without homeostatic function, with adverse consequences, and as ways to relieve stress, fits some, but not all people with excess weight. Furthermore, we did not include studies on the complete spectrum of eating disorders that may include features of compulsive eating (e.g., BE/purging type Anorexia Nervosa (AN) or Other Specified Feeding or Eating Disorders, Purging Disorder, or Night Eating Syndrome). Nevertheless, our inclusion of disorders is in line with recent reviews on compulsive behavior as a central feature of certain eating disorders (i.e., BED), obesity, and the emerging concept of FA (Moore et al., 2017). In addition, this review focused only on the potential shared cognitive processes, and hence, whether there are overlapping neural and behavioral processes related to compulsivity across the spectrum of eating-related issues is yet to be determined. Importantly, the four cognitive domains of compulsivity are proposed to have distinct neural correlates. Although it was beyond the scope of the current review, future studies should aim to examine the neural underpinnings of the cognitive domains in an eating context.

Finally, we consider the practical relevance of these findings, including consideration of how compulsivity has typically been examined in the eating domain and the limitations of such methodological approaches. First, the cognitive tasks used in the reviewed studies have been borrowed from other fields, and thus, some tasks were used to measure multiple constructs (i.e., inhibition and set-shifiting) or were not clearly operationalized in the context of compulsivity. Thus, future studies should use cognitive tasks specificially developed to measure the different components of compulsivity. Second, most of the reviewed studies examined group differences (i.e., clinical vs. healthy controls) in compulsivity-related cognitive performance. However, few studies investigated the relationship between performance on cognitive tasks and compulsive behavioral tendencies. Thus, future studies should include self-report questionnaires measuring phenotypic descriptions of compulsive behavior, including the Obsessive Compulsive Eating Scale (Niemiec et al., 2016) or the Creature of Habit Scale (Ersche et al., 2017).

In addition, there was a lack of experimental studies on compulsivity-related cognitive drivers of FA, despite its emerging conceptualization as a disorder characterized by compulsive eating behavior (Davis, 2017). Therefore, it is unclear whether so-called FA shares overlapping impairments in compulsivity-related cognitive functioning with BN, BED and obesity. Indeed, most of the research on FA has focused on the clinical symptoms as measured with the YFAS; however, some recent studies have recently reported impaired impulsive action (i.e., go/no-go responses; Meule et al., 2012) and choice (i.e., delay discounting; VanderBroek-Stice et al., 2017) in FA. Future studies should examine compulsivity-related cognitive processing in FA to determine whether it is similarly characterized by such deficits.

A further limitation of the reviewed literature is that the studies relied heavily on cross-sectional rather than longitudinal designs. Therefore, the chronology of the cognitive components driving compulsivity in eating-related populations remains unclear. Specifically, cognitive performance deficits may be linked to the development and maintenance of compulsive eating behavior, and in turn, eating-related conditions. For example, it may be that an inefficient ability to adapt behavior after negative feedback or greater attentional engagement toward food cues confers increased risk of developing compulsive eating. Alternatively, these deficits may be a consequence of compulsive eating and as such, linked to the prognosis of the eating-related conditions and treatment outcomes. We hypothesize that this is likely a dynamic process in which there are trait vulnerabilities to develop compulsive eating behavior that are then exacerbated through reinforcement and maladaptive learning mechanisms. Future prospective and longitudinal studies should examine whether compulsivity is a vulnerability factor, which predates the development of obesity or eating disorders, or whether it overlaps with the onset of clinical symptoms, or both. It is also important to determine whether problematic eating behavior reflects a transition from impulsivity to compulsivity, as has been proposed in addiction models (Everitt and Robbins, 2016). Further to this point, the current review focused on studies that examined compulsivity-related cognitive processes, so we did not review evidence for impulsivity-related cognitive processes. Thus, it is not clear how cognitive processes underlying impulsivity and compulsivity are related in the context of eating-related behaviors, or how they might interact with other processes such as decision-making.

Based on the aforementioned limitations, we make several recommendations for future research. First, future studies should examine all four compulsivity-related cognitive components within the same study in a particular population (e.g., patients with BED), rather than examining only discrete components. In parallel, research should examine these four components trans-diagnostically in the context of eating-related issues, which would allow us to determine whether there are shared underlying mechanisms driving compulsive eating behavior across disorders. Furthermore, some of the cognitive processes reviewed (i.e., set-shifting and reversal learning) are sub-components of the higher-order construct, cognitive flexibility (Wildes et al., 2014). Therefore, it would be useful to measure both of these sub-components in a single study to determine whether they interact in predicting compulsive behavior based on the proposed separate neural circuitry (Fineberg et al., 2014). Importantly, examining compulsivity-related cognitive processes at different stages of eating-related issues using prospective or longitudinal designs would enable the prediction of vulnerability to compulsive eating behavior. In addition, longitudinal research would have implications for informing the development of transdiagnostic prevention and treatment strategies designed to improve cognitive functioning, which may be a promising avenue for reducing compulsive behavioral tendencies across a range of disorders.

## Conclusion

The findings of some of the included studies support the notion that impairments in compulsivity-related cognitive components may characterize a range of eating-related conditions, although the evidence was inconsistent or lacking for some disorders. The mixed findings in most domains likely resulted from divergent cognitive assessment tasks and possible interactions with dietary restraint, anxiety/stress, and emotional eating. Future research should comprehensively examine the cognitive components of compulsivity, include measures of compulsive eating, and use longitudinal designs to inform the clinical prediction of compulsivity-related symptoms and the development of interventions for compulsive eating.

## Author Contributions

NK and AV-G contributed to the conceptualization of the review. NK wrote the first draft of the manuscript. NK, EA and AV-G wrote sections of the manuscript. All authors contributed to manuscript revision, read and approved the submitted version.

## Conflict of Interest Statement

The authors declare that the research was conducted in the absence of any commercial or financial relationships that could be construed as a potential conflict of interest.
