# Erratum: The Cognitive Drivers of Compulsive Eating Behavior

**DOI:** 10.3389/fnbeh.2019.00230

**Published:** 2019-10-01

**Authors:** Frontiers Production Office

**Affiliations:** Frontiers Media SA, Lausanne, Switzerland

**Keywords:** compulsivity, cognitive functioning, eating behavior, obesity, bulimia nervosa, binge eating, food addiction

Due to a typesetting error, the citations for Janssen et al. (2017) were removed from the final version. The citations should have appeared in the **Attentional Bias/Disengagement** section, paragraph two, “…(body Mass index, BMI and abdominal fat; Janssen et al., 2017)”, in the **Habit Learning** section, paragraph two, “…which suggests that these two systems are unbalanced (Horstmann et al., 2011; Janssen et al., 2017).”, and in the **Discussion** section, paragraph five, “…outcome devaluation *via* satiation (Horstmann et al., 2011; Janssen et al., 2017).”

The publisher apologizes for this error. The original article has been updated.
